# Single-Wavelength Visible-Light-Induced Reversible Isomerization of Stiff-Stilbene under Dynamic Covalent Control

**DOI:** 10.1021/acs.orglett.5c00707

**Published:** 2025-03-31

**Authors:** Indigo M. Bekaert, Maxime A. Siegler, Sander J. Wezenberg

**Affiliations:** † Leiden Institute of Chemistry, Leiden University, Einsteinweg 55, 2333 CC Leiden, The Netherlands; ‡ Department of Chemistry, Johns Hopkins University, 3400 North Charles Street., Baltimore, Maryland 21218, United States

## Abstract

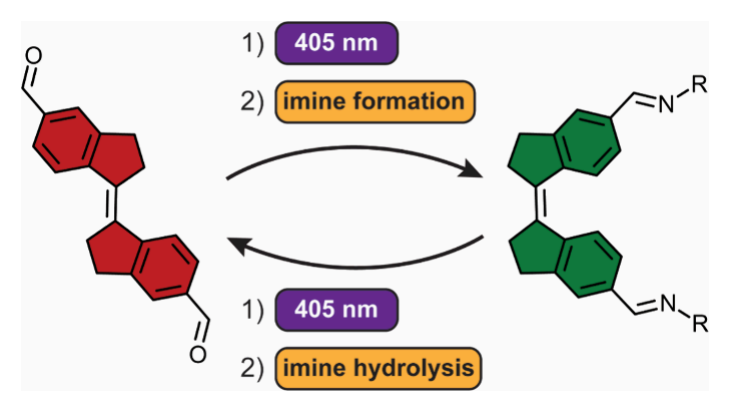

Formylation of stiff-stilbene enables isomerization by visible instead of UV light, as is monitored by UV–vis and ^1^H NMR spectroscopy. Further, it allows dynamic covalent imine formation, which gives rise to slightly blue-shifted absorbance. As a result, the irradiation wavelength that is used to promote *E* → *Z* isomerization for the formylated species, causes the opposite *Z* → *E* isomerization for the imine derivative. This feature can be used to produce either isomer with the same light source under control of imine formation and hydrolysis. Single crystal X-ray analysis and TD-DFT calculations provide structural and electronic insight.

Molecular photoswitches have been used to control a myriad of chemical and biological functions.^1,2^ Under light irradiation, they interconvert between states with different absorption spectra (i.e., photochromism), and hence, by changing wavelength, either forward or backward isomerization can be induced. The use of light as stimulus has the key advantage that it can be applied with high spatiotemporal precision and, where many photoswitches originally had the drawback that they required use of harmful UV light, several strategies are nowadays available to red-shift their absorption to the visible-light range.^3^

An important challenge is to gain additional control over the light-induced isomerization process by other, orthogonal stimuli, which enables development of systems with more sophisticated and complex behavior.^4^ Yet, while the properties of some photoswitches, in particular diarylethene and azobenzene, have been modulated by chemical stimuli,^5^ this was mostly used for gating, i.e., to turn photoswitching “ON/OFF”. Reverting the isomerization direction—under single wavelength irradiation—using chemical stimuli could be key to achieving ratcheting^6^ and autonomous behavior, but so far has only been demonstrated by our group for *N*-heterocyclic hemi-indigo,^7^ in which (de)protonation breaks and reforms intramolecular hydrogen bonds that inhibit photoisomerization. Other methods to control photoswitching properties by chemical stimuli, beyond protonation and host–guest binding, are still highly desired.

We took inspiration from the mammalian visual transduction cycle, in which dynamic imine bond formation between 11-*cis*-retinal and the opsin protein is essential.^8^ Here, incident visible light causes the isomerization of the retinal Schiff base to the all-*trans* form, which is then hydrolyzed (and retinal isomerized back to the 11-*cis* form enzymatically). This imine hydrolysis and reformation significantly alters the absorbance properties of retinal, which is crucial for its highly efficient photoisomerization. By analogy, we envisioned modulating the UV–vis absorbance of an artificial molecular photoswitch through dynamic covalent imine bond formation.^9^ Our photoswitch of choice was stiff-stilbene, i.e. a fused five-membered ring analog of stilbene, which is increasingly applied in various research areas.^10,11^ Where this type of molecular photoswitch is usually isomerized by UV light, we have shown visible-light isomerization *via* the introduction of push–pull substituents,^5j^ whereas the group of Feringa recently achieved this through *ortho*-formylation of stiff-stilbene-6,6′-diol.^12^ We predicted that transformation of formyl groups into imines, when located in the *para*-position with respect to the central olefinic bond, would affect the electronic properties and, hence, the UV–vis absorbance. As a result, imine bond formation and hydrolysis could enable bidirectional photoswitching under irradiation with a single light source.

Herein, we describe diformylated stiff-stilbene **1** (Scheme 1), which can be isomerized between *E* and *Z* forms using solely visible light (405 nm/455 nm). Condensation with an amine affords diimine-derivative **2**, of which the absorption is blue-shifted with respect to **1**. Where for the diformylated compound, 405 nm irradiation leads to enrichment in *Z*-isomer, conversely, the diimine derivative is isomerized to the *E*-isomer with this wavelength. This feature allows to address either isomer by the same wavelength of light, under control of dynamic covalent bond formation. To our knowledge, this work represents the first example of dynamic covalent chemistry-controlled photoisomerization. It is expected to provide a stepping stone toward more complex—and possibly fully autonomous—light-driven functional molecular systems.

**Scheme 1:**
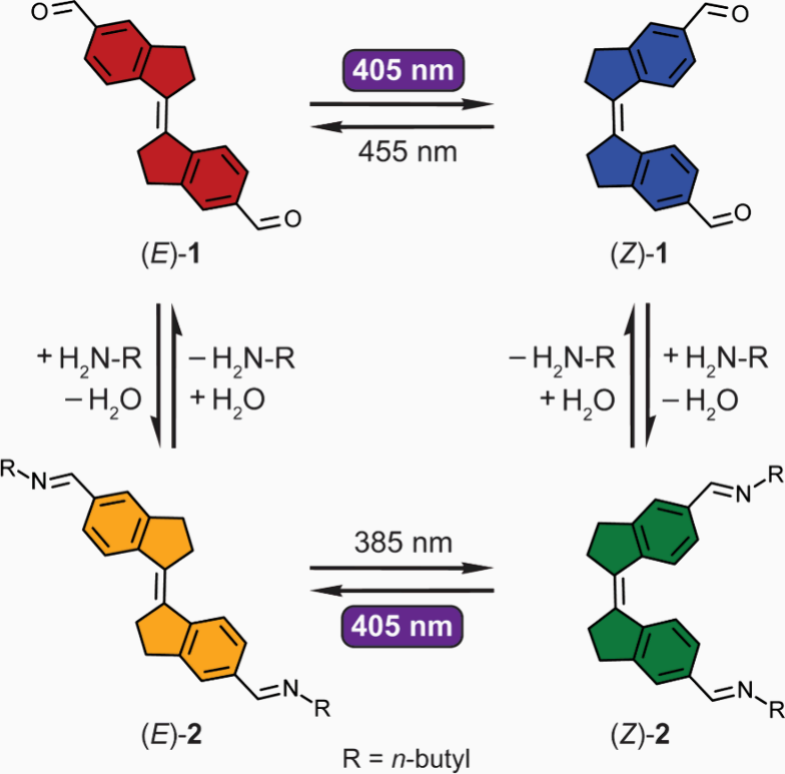
Dynamic Covalent Chemistry-Controlled Photoisomerization Cycle of Diformylated Stiff-Stilbene, **1**

Stiff-stilbene (*Z*)-**1** was synthesized through formylation of its previously reported, parent dibromo-derivative (*Z*)-**3**^5j^ using *n*-butyllithium, where DMF acted as the carbonyl surrogate (see Supporting Information (SI) for synthetic details and characterization and Scheme S1). The respective *E*-isomer could not be obtained by using the same method due to the limited solubility of the (lithiated) precursor. Therefore, all experimental studies described herein were performed by starting with the *Z*-isomer.

The effect of irradiating (*Z*)-**1** was first examined with UV–vis spectroscopy in a CH_3_CN solution (Figure 1a). In line with what was recently reported for stiff-stilbene containing aldehyde groups in *para*-position to the central olefinic bond,^12^ the absorbance of (*Z*)-**1**, showing a maximum at λ = 382 nm, is significantly red-shifted compared to unfunctionalized stiff-stilbene (λ_max_ = 330 nm).^13^ Irradiation on the shoulder band at 455 nm resulted in a hypsochromic shift and increase of the overall absorption, which is indicative of *Z* → *E* isomerization.^10,11^ When no further spectral changes were noted; i.e., the photostationary state (PSS) was reached, the absorbance maxima were located at λ = 375 nm and 394 nm. Upon consecutive irradiation with 405 nm light, the spectral changes were reversed, revealing (partial) recovery of the *Z*-isomer. Subsequent irradiation with shorter wavelengths (in the UV-region) led to more *E* → *Z* back-isomerization. For all wavelengths used, irradiation was halted when the photostationary states had been reached and a clear isosbestic point was observed at λ = 406 nm, indicating unimolecular conversion. Further, the isomerization cycle could be repeated multiple times using 455 nm/405 nm visible-light without major signs of fatigue (Figure S5).

**Figure 1:**
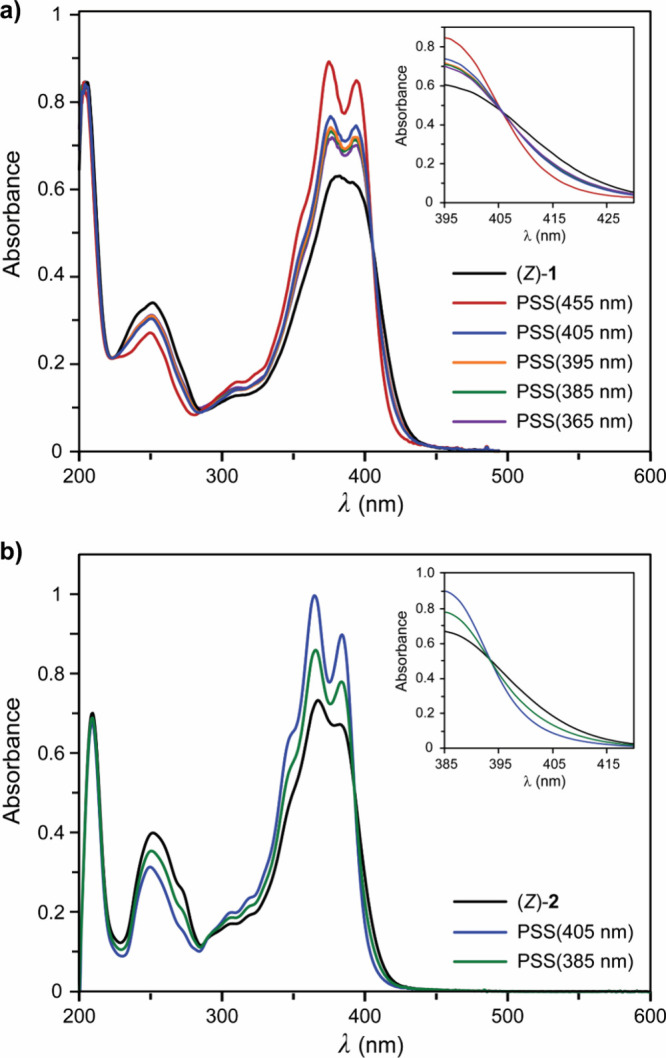
(a) UV–vis spectra of (*Z*)-**1** (2.0 × 10^–5^ M in dry, degassed CH_3_CN) upon irradiation with 455 nm, 405 nm, 395 nm, 385 nm, and 365 nm light until PSS was reached. (b) UV–vis spectra of (*Z*)-**2** (2.0 × 10^–5^ M in dry, degassed CH_3_CN) upon irradiation with 405 nm and 385 nm light until PSS was reached.

Next, ^1^H NMR spectroscopy was used to determine the PSS ratios obtained upon visible-light-induced isomerization. When a solution of (*Z*)-**1** in CD_3_CN was first irradiated with 455 nm, a new set of ^1^H NMR signals appeared, which were assigned to (*E*)-**1** (Figure S6). By relative integration of both signal sets after PSS_455_ had been reached, a *Z*/*E*-ratio of 8:92 was calculated. By subsequent irradiation with 405 nm light, the relative integral of the signal set assigned to the *Z*-isomer increased and the *Z*/*E*-ratio at PSS_405_ was determined as 41:59, in line with what was observed by UV–vis spectroscopy. The PSS ratios at the UV irradiation wavelengths were calculated using the change in absorbance at λ = 382 nm and the PSS_455_ ratio determined by ^1^H NMR spectroscopy, showing that up to 66% of (*Z*)-**1** can be regenerated with 365 nm light (Table S1). Importantly, the ^1^H NMR integral ratio remained unchanged when a 50:50 mixture of (*Z*)-**1** and (*E*)-**1** in CD_3_CN was left for 7 days at room temperature (Figure S7), indicating a high thermal stability, as is characteristic for stiff-stilbene.^14^

X-ray crystallographic analysis of single crystals obtained by slow evaporation of an irradiated solution of (*Z*)-**1** in chloroform unequivocally confirmed photogeneration of (*E*)-**1**.^14^ Its solid-state structure (Figure 2) is fully planar with a central C_Ar_–C=C–C_Ar_ dihedral angle of 180°, similar to previously reported structures of unsubstituted stiff-stilbene.^13,15^ Worthy to note is that both aldehyde substituents are in plane with the aromatic core to extend the conjugated π-system, which is most likely the origin of the red-shifted absorbance of **1**.

**Figure 2:**
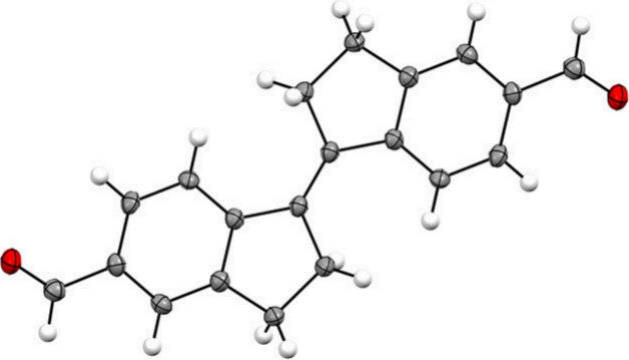
Displacement ellipsoid plot (50% probability level) for (*E*)-**1** as found in the crystal structure at 110(2) K.

TD-DFT calculations at the B3LYP/6-311G++(d,p) level of theory, using an IEFPCM CH_3_CN solvation model, revealed a smaller optical gap with respect to unsubstituted stiff-stilbene (Figures S11 and S13), supporting the more red-shifted absorbance.^11f^ For (*Z*)-**1**, λ_max_(calc) was found at 418 nm, whereas for (*E*)-**1**, λ_max_(calc) was located at 412 nm, which is in reasonable agreement with experimental data.^16^ In both cases, the S_0_ → S_1_ excitation was recognized as a pure HOMO–LUMO π–π* transition. The LUMO displayed a clear antibonding character at the central olefinic bond, illustrating a decrease in double bond character in the excited state.

We then studied the effect of imine formation on the absorbance and photoisomerization properties. The diimine derivative (*Z*)-**2** was formed by condensation of the aldehyde groups with excess *n*-butylamine in CD_3_OD and full conversion was corroborated by ^1^H NMR spectroscopy (Figure S8). The equilibrium between an imine and its precursors is known to depend on the chosen solvent,^17^ among others, and in CD_3_CN we did not observe the formation of (*Z*)-**2**. Moreover, the compound was not stable upon isolation. Therefore, to carry out UV–vis absorption measurements, a portion of the NMR sample in CD_3_OD was diluted in CH_3_CN (0.18% (v/v) CD_3_OD/CH_3_CN). As is shown in Figure 1b, imine formation causes a blue-shift in the absorbance; i.e., a maximum is now observed at 368 nm compared to 382 nm for dialdehyde (*Z*)-**1**. Interestingly, where for the dialdehyde derivative 405 nm irradiation resulted in isomerization back to the *Z*-isomer, in this case, the highest *Z* → *E* conversion was attained with this wavelength. When PSS_405_ was reached, the absorbance maxima were located at λ = 366 nm and 385 nm. The spectral changes could now be reversed by irradiation with 385 nm light, again demonstrating reversibility of the isomerization process. Further, consecutive irradiation with 405 nm and 385 nm showed good photofatigue resistance of the imine adduct (Figure S9). The isosbestic point maintained during irradiation hints at a unimolecular isomerization reaction and confirms the stability of the imine bond during photoisomerization. The PSS ratios could not be determined by ^1^H NMR spectroscopy in neither this solvent mixture nor CD_3_CN due to the instability of the imine bonds but, nevertheless, they could be quantified in CD_3_CN/CD_3_OD (3:1 (v/v)) (*vide infra*).

Also for diimine derivative **2**, TD-DFT calculations [B3LYP/6-311G++(d,p), IEFPCM CH_3_CN] were performed, to support the observed spectral blue-shift relative to diformylated precursor **1** (Figures S12 and S13). As expected, an increase in the optical gap for **2**, as compared to **1**, was observed, and the 14 nm hypsochromic shift that was found experimentally was seen in the computed spectra as well. Furthermore, the calculated absorbance maxima are in reasonable agreement with the experimental ones [λ_max_((*Z*)-**2**, calc) = 405 nm, λ_max_((*E*)-**2**, calc) = 398 nm] and again, the S_0_ → S_1_ excitation was a pure HOMO–LUMO π–π* transition for both *Z*- and *E*-isomer with strong antibonding character at the central olefin in the excited state.^16^

The fact that for dialdehyde **1** 405 nm irradiation leads to enrichment in the *Z*-isomer, while for diimine **2** it results in conversion to the *E*-isomer, means that reversible isomerization can be achieved using the same light source, under control of dynamic covalent bond formation. Hence, we hypothesize that under thermodynamic equilibrium conditions [where there is constant exchange between (*E*)-**1** and (*E*)-**2** as well as between (*Z*)-**1** and (*Z*)-**2**], forward isomerization is dominant in the dialdehyde form, while backward isomerization preferably takes place in the diimine form. This would lead to a net directional cycle (Scheme 1), which continuously occurs under 405 nm irradiation at the photostationary state. However, since under such steady-state conditions it is not possible to monitor the individual photoconversion and imine formation/hydrolysis steps, we chose to perform one full cycle in which the equilibrium was pushed fully to **1** and **2** using acetic acid and excess *n*-butylamine, respectively.

This chemically controlled, single-wavelength-induced isomerization cycle was demonstrated *in situ* by ^1^H NMR spectroscopy. To start this cycle with *E* → *Z* isomerization induced by 405 nm light, first, a solution enriched in (*E*)-**1** (90%) was generated by 455 nm irradiation of (*Z*)-**1** in CD_3_CN/CD_3_OD (3:1 (v/v)) (Figures 3 and S10). Irradiation of this solution with 405 nm light led to an increase in the relative integrals of ^1^H NMR signals belonging the *Z*-isomer, and a *Z*/*E* ratio at the PSS of 30:70 was calculated. Next, an aliquot of *n*-butylamine was added to initiate imine formation, and over time, a loss of aldehyde signals around 10 ppm was seen, whereas a new set of signals appeared. In particular, the new peaks at 8.33 ppm and 8.31 ppm belonging to newly formed imine groups are indicative of complete formation of (*E*)-**2** and (*Z*)-**2** respectively. Importantly, the *E*/*Z* isomer ratio did not change over the time span it took to complete conversion to the diimine derivative (2 days), showing that the high thermal stability of stiff-stilbene is preserved. The mixture was then irradiated again with 405 nm, where this now increased the relative integrals of the ^1^H NMR signals of the *E*-isomer, to obtain a new *Z*/*E*-ratio of 10:90. By addition of an aqueous solution of acetic acid to promote imine hydrolysis, the signals belonging to **2** diminished and the original signals of **1** were recovered, completing the cycle.

**Figure 3:**
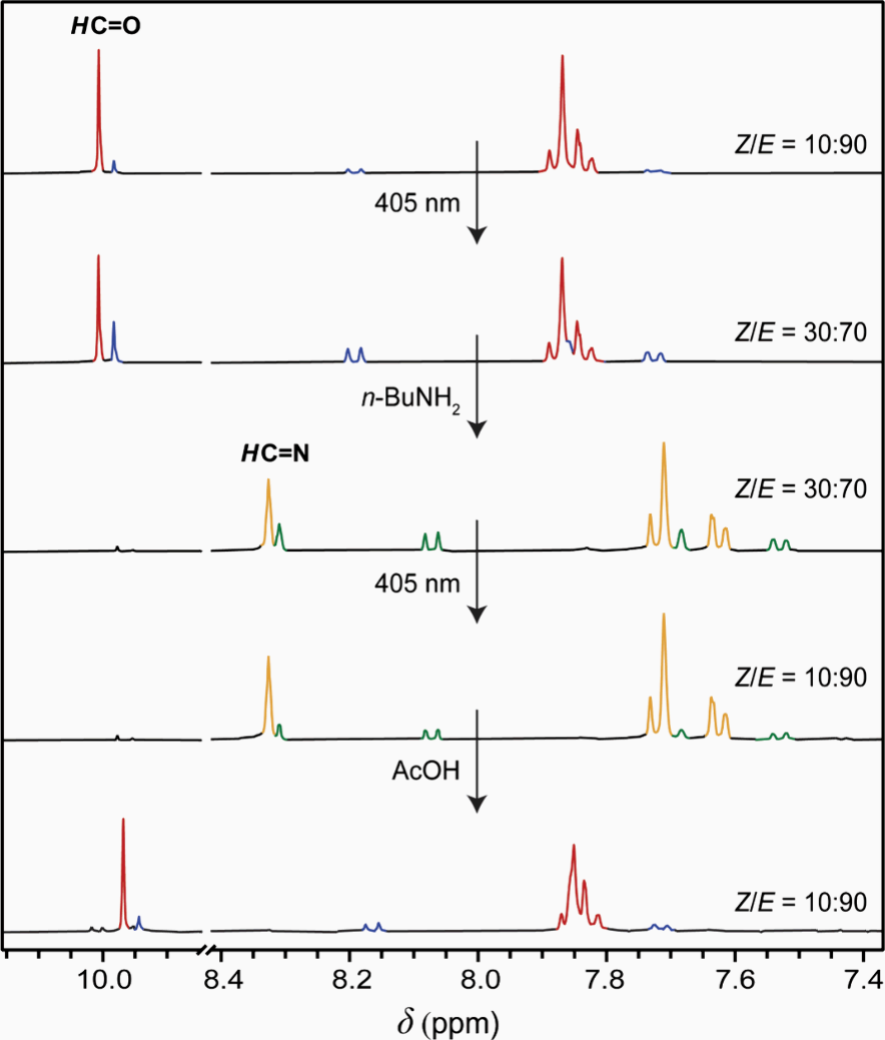
^1^H NMR spectra of the chemically controlled photoisomerization cycle using light of one wavelength (see Scheme 1 for color coding and Figure S10 for full ^1^H NMR spectra and details).

In summary, we have prepared 5,5′-dialdehyde-substituted stiff-stilbene and shown that it can undergo *E*/*Z* isomerization using visible light (405 nm/455 nm). When the aldehyde groups are transformed into imines upon condensation with *n*-butylamine, the UV–vis absorbance is significantly blue-shifted, and therefore, back and forth isomerization was achieved by the use of shorter wavelengths (385 nm/405 nm). That is, where for the dialdehyde derivative 405 nm irradiation was used to regenerate the *Z*-isomer, the same wavelength induced isomerization of the diimine-substituted compound to the *E*-isomer. Owing to this opposite isomerization direction under irradiation by the same wavelength, a dynamic covalent chemistry-controlled photocycle was envisioned, in which either the *E-* or *Z-*stiff-stilbene isomer can be addressed by imine formation and hydrolysis, respectively. To the best of our knowledge, this work shows the first example of reversible photoswitching with the same source of light, under control of a chemical stimulus other than (de)protonation.^7^ We expect it to lead to dynamic functional molecular systems with increased levels of control and autonomous behavior.

## Data Availability

The data underlying this study are available in the published article and its Supporting Information Statement.

## Supporting Information Available

Experimental procedures and characterizations by ^1^H, ^13^C{^1^H} APT, ^1^H–^1^H COSY, and ^1^H–^13^C HSQC NMR, and IR (ATR), UV–vis, and HRMS; TLC retention factor; and melting point analysi;, photoisomerization and imine-formation studies; TD-DFT calculations; single crystal X-ray analysis (CCDC 2400457) (PDF)
