# Supplementary material for: Single-Wavelength Visible-Light-Induced Reversible Isomerization of Stiff-Stilbene under Dynamic Covalent Control
Source: Org Lett. 2025 Mar 31;27(14):3612–6. doi: 10.1021/acs.orglett.5c00707 (PMC11998061; doi:10.1021/acs.orglett.5c00707)
Supplement: Supplementary file 1 — ol5c00707_si_001.pdf [file ol5c00707_si_001.pdf]

SUPPORTING INFORMATION

Single-Wavelength Visible-Light-Induced Reversible Isomerization of Stiff-Stilbene under Dynamic Covalent Control

Indigo M. Bekaert,[a] Maxime A. Siegler,[b] and Sander J. Wezenberg*[a]

[a] *Leiden Institute of Chemistry, Leiden University, Einsteinweg 55, 2333 CC Leiden, The Netherlands*

[b] *Department of Chemistry, Johns Hopkins University, 3400 North Charles St., Baltimore, MD 21218, United States*

Email: s.j.wezenberg@lic.leidenuniv.nl

Table of Contents

Experimental section …………………………………………………………………….. S2

^{1}H and ^{13}C NMR spectra of title compound …………………………………………… S4

UV-vis photoisomerization studies of (*Z*)-**1** …………………………………………….. S6

^{1}H NMR photoisomerization study of (*Z*)-**1** ..…………………………………………… S7

^{1}H NMR thermal stability study of **1** ………………………………………………….. S8

^{1}H NMR in situ formation study of (*Z*)-**2** ……………………………………………….. S9

UV-vis photoisomerization study of (*Z*)-**2** ……………………………………………… S10

^{1}H NMR study of chemically-controlled photoisomerization …………………………... S11

Single crystal X-ray crystallography ……………………………………………………... S12

Time-dependent DFT calculations ……………………………………………………… S14

References …………………………………………………………………………….. S18

Experimental section

General methods and materials:

THF and CH_3CN were dried using a Pure Solve 400 solvent purification system from Innovative Technology. Dry DMF was purchased from Acros Organics. Stiff-stilbene (*Z*)-**3** was prepared according to a procedure reported in the literature.[1] All other chemicals and solvents were commercial products and were used without further purification. The degassing of the solvents was carried out by purging with N_2 for 15 min unless stated otherwise. Column chromatography was performed using silica gel (SiO_2) purchased from Screening Devices BV (pore diameter 55-70 Å, surface area 500 m^2g^{-1}). Thin-layer chromatography (TLC) was carried out on aluminum sheets coated with silica 60 F254 obtained from Merck. Compounds were visualized with UV light (254 nm). ^1H and ^{13}C NMR spectra were recorded on Bruker AV 400 and Bruker AV 600 at 298 K. CD_3OD, CD_3CN and $CDCl_3$ were purchased from Eurisotop. Chemical shifts (δ) are denoted in parts per million (ppm) relative to residual protiated solvent (CD_3OD: for ^1H detection, δ = 3.31 ppm, CD_3CN: for ^1H detection, δ = 1.96 ppm, $CDCl_3$: for ^1H detection, δ = 7.26 ppm; for ^{13}C detection, δ = 77.16 ppm). The splitting pattern of peaks is designated as follows: s (singlet), d (doublet), t (triplet) m (multiplet). Structural assignments were made with additional information from gCOSY and gHSQC experiments. Infrared spectra were recorded on a PerkinElmer FT-IR Spectrum Two spectrometer using an ATR unit. Absorbance bands are reported in wavenumbers (v) which are in units of reciprocal centimeters (cm^{-1}) and the intensities are designated as follows: s (strong), m (medium), w (weak), and sh (shoulder). Melting points were determined with a Büchi M560 apparatus. High-resolution mass spectrometry (ESI-MS) was performed on a Thermo Scientific Q Exactive HF spectrometer with electron spray ionization. UV-vis spectra were recorded on an Agilent Cary 8454 spectrometer using 1 cm quartz cuvettes at 20 °C. Irradiation of UV-Vis and NMR samples was carried out using Thorlab model M365F1 (3.00 mW), M385F1 (9.0 mW), M395F3 (4.8 mW), M405F1 (3.0 mW), M455F3 (17 mW) LEDs positioned at a distance of 1 cm to the sample.

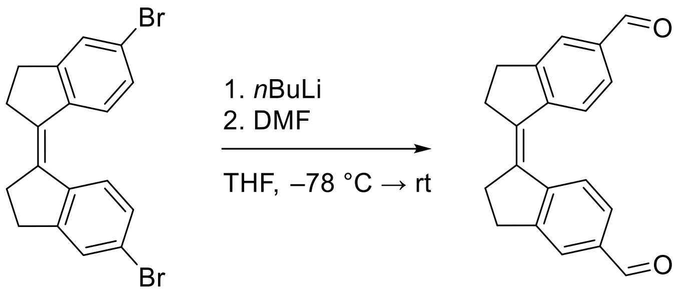

(*Z*)-**3** (*Z*)-**1**

Scheme S1. Synthesis of diformylated stiff-stilbene (Z)-**1**.

(*Z*)-2,2',3,3'-tetrahydro-[1,1'-biindenylidene]-5,5'-dicarbaldehyde [(*Z*)-1]:

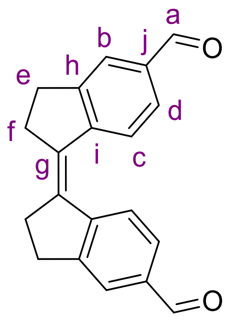

Compound (*Z*)-**3** (94 mg, 0.24 mmol) was dissolved in dry, degassed THF (15 mL) under N_2 atmosphere in the dark. The solution was cooled to –78 °C and *n*BuLi (1.6 M in hexanes, 0.33 mL, 0.53 mmol) was added dropwise over 10 minutes. After 1 h of stirring at –78 °C, dry DMF (0.20 mL, 2.4 mmol) was added and the solution was allowed to warm to rt and further stirred overnight. The mixture was then cooled to 0 °C and treated with H_2O (30 mL), followed by extraction with CH_2Cl_2 (3 x 50 mL). The combined organic phases were dried over Na_2SO_4, filtered and concentrated *in vacuo* at 30 °C. Purification by column chromatography (SiO_2, CH_2Cl_2) afforded product (*Z*)-**1** (13 mg, 18%) as an orange solid.‡ R_f = 0.29 (SiO_2, CH_2Cl_2); m.p. 177-179 °C; ^{1}H NMR (400 MHz, $CDCl_3$): δ = 9.99 (s, 2H; H_a), 8.14 (d, J = 8.04 Hz, 2H; H_d), 7.82 (s, 2H; H_b), 7.69 (d, J = 8.04 Hz, 2H; H_c), 3.11 – 3.03 (m, 4H; H_e), 2.94 – 2.86 (m, 4H; H_f) ppm; ^{13}C NMR (101 MHz, $CDCl_3$, assignment is based on a HSQC spectrum): δ = 191.8 (C_a), 149.7 (C_h), 146.4 (C_i), 138.3 (C_j), 135.9 (C_g), 128.6 (C_c), 126.3 (C_b), 123.7 (C_d), 35.3 (C_f), 30.4 (C_e) ppm; IR (ATR): ν = 2921 (w), 2833 (w), 2787 (w), 2726 (w), 1686 (sh), 1673 (s), 1601 (s), 1560 (s), 1443 (m), 1428 (m), 1380 (m), 1339 (w), 1322 (w), 1304 (m), 1281 (m), 1243 (s), 1232 (s), 1185 (m), 1145 (m), 1106 (w), 1087 (m) cm^{-1}; UV-vis (CH_3CN): λ_{max} (ε) = 382 (25200), 251 (15000 $M^{-1}cm^{-1}$); HRMS (ESI) m/z: 289.1223 ([M+H]+, calcd for $C_{20}H_{17}O_2^+$: 289.1223).

‡ Please note that double formylation through this method is generally low yielding,[2,3] however alternative routes either require more steps, or use reagents that are incompatible with the stability of the stiff-stilbene central double bond.[4–6]

^{1}H and ^{13}C NMR spectra of title compound

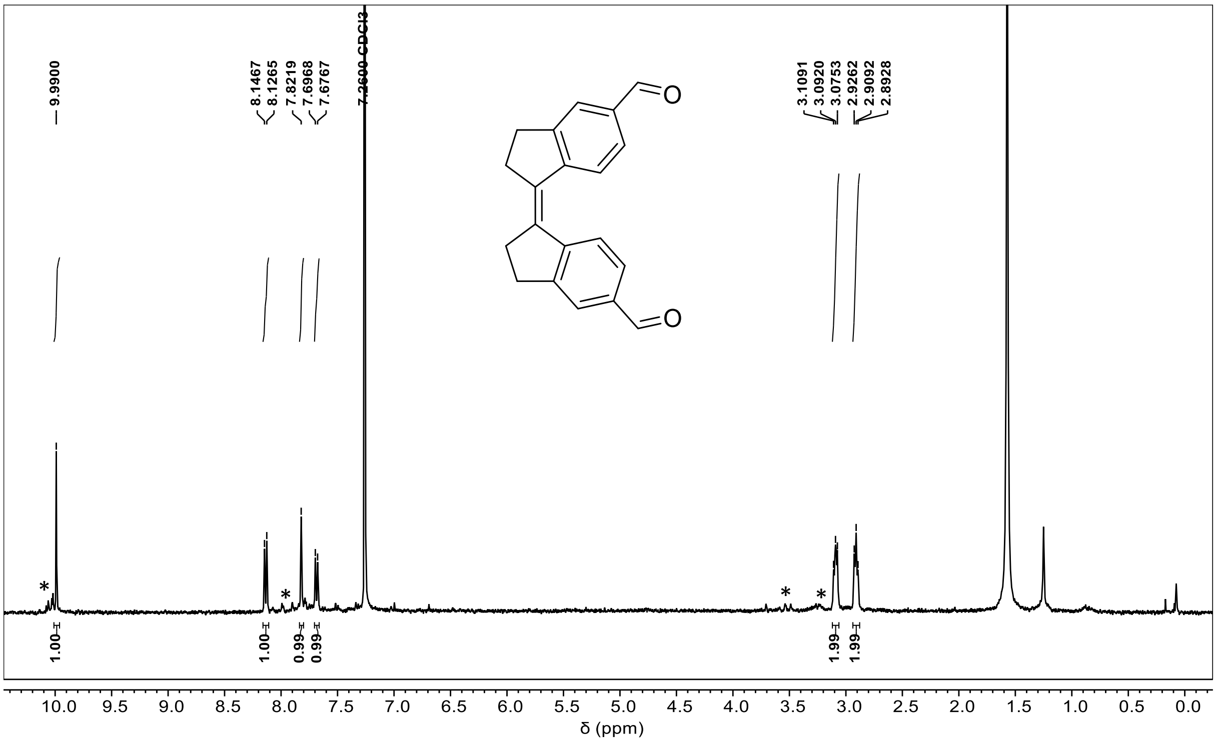

Figure S1. ^{1}H NMR spectrum of (*Z*)-**1** in $CDCl_3$ (400 MHz). The trace impurity (<5%), indicated with (*) is (*E*)-**1**, which formed during work-up in dichloromethane (and over time in chloroform) and could not be fully separated by column chromatography.

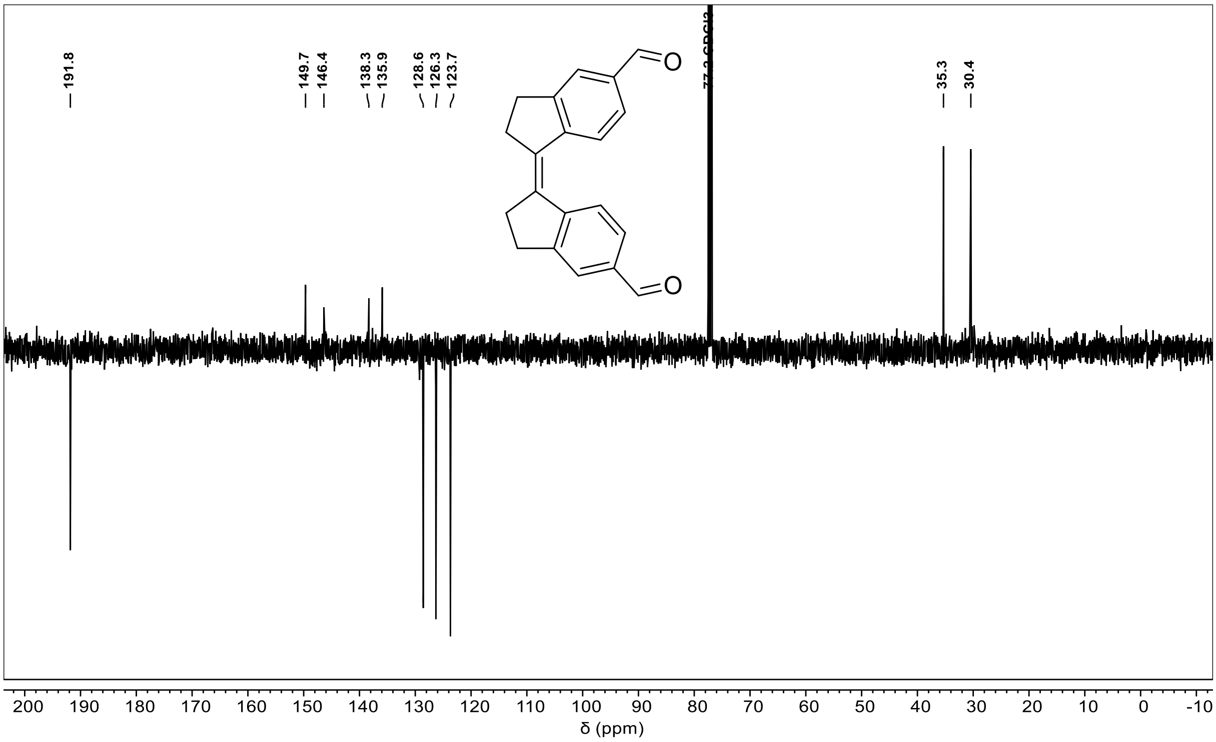

Figure S2. $^{13}C\{^{1}H\}$-APT NMR spectrum of (*Z*)-**1** in $CDCl_3$ (101 MHz).

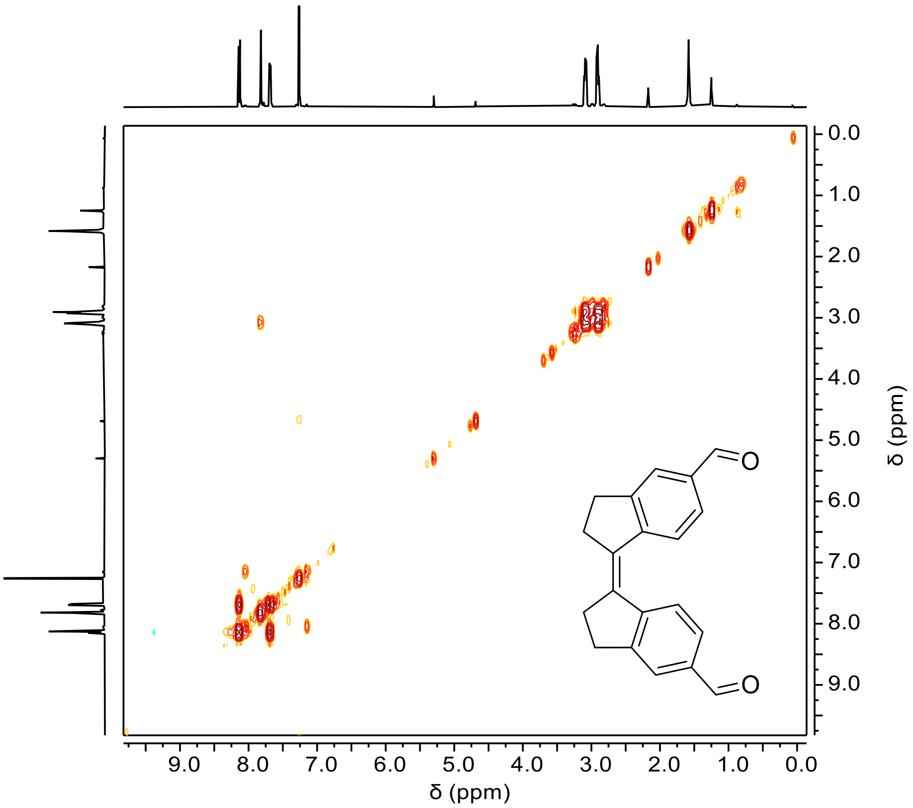

Figure S3. ^{1}H-^{1}H COSY spectrum of (*Z*)-**1** in $CDCl_3$ (101 MHz).

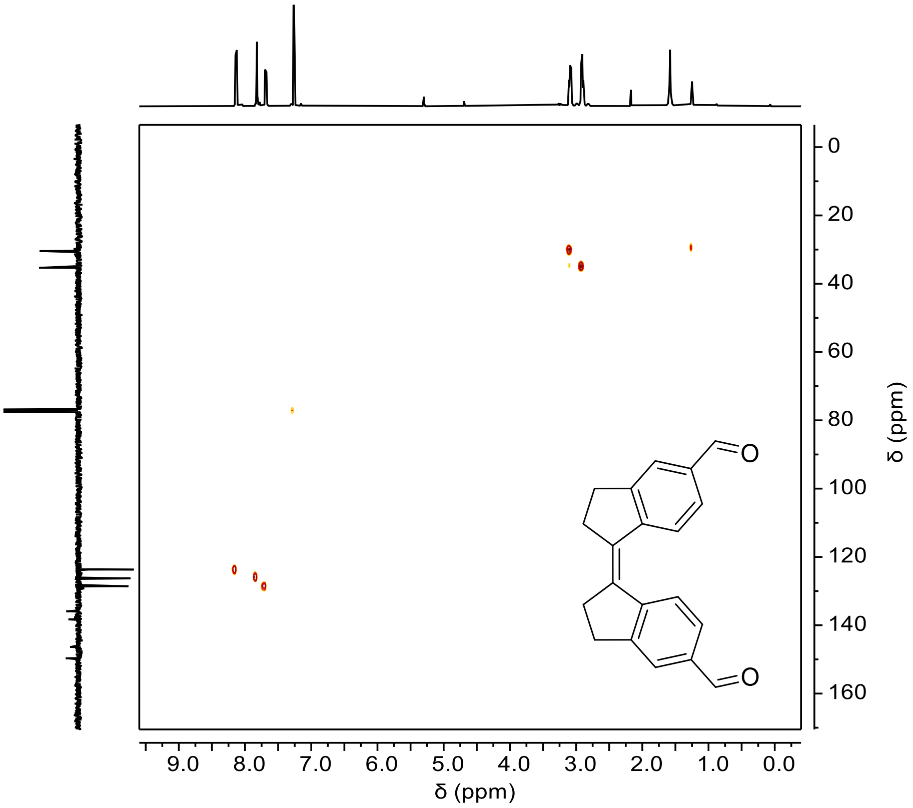

Figure S4. ^{1}H-^{13}C HSQC spectrum of (*Z*)-**1** in $CDCl_3$ (101 MHz).

UV-vis photoisomerization studies of (*Z*)-1

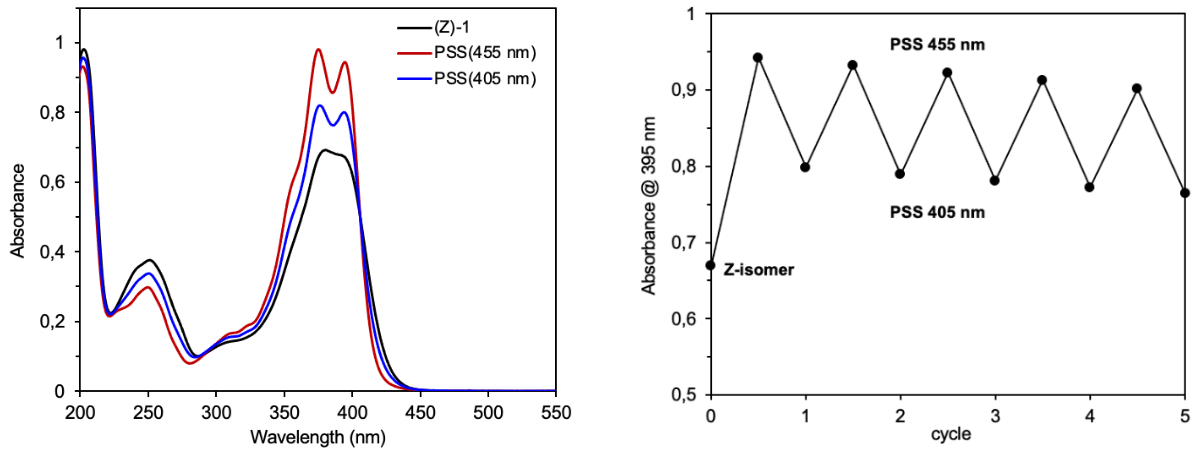

Figure S5. (left) UV-vis absorption spectra of (*Z*)-**1** (2.5 x 10^{-5} M in dry, degassed CH_3CN) upon irradiation with 455 nm and 405 nm light and (right) absorbance intensity plot (at λ = 395 nm) upon sequential irradiation with 455 nm (7 min) and 405 nm (20 s); After five 455/405 nm irradiation cycles, 4% loss in absorbance intensity was observed.

Table S1. PSS ratios estimated using UV-Vis absorption and ^{1}H NMR (PSS_{455}) data.[a]

	Abs (382 nm)	Δ Abs (382 nm)	x_E (%)	PSS (*Z*/*E*)
(*Z*)-**1**	0.63	-	0	-
PSS_{455}	0.80	0.17	92	8:92
PSS_{430}	0.80	0.17	91	9:91
PSS_{405}	0.74	0.11	58	42:58[b]
PSS_{395}	0.71	0.08	42	58:42
PSS_{385}	0.70	0.07	40	60:40
PSS_{365}	0.69	0.06	34	66:34

a Calculated using the PSS_{455} value determined by ^{1}H NMR spectroscopy (see Figure S6) and the absorption at λ = 382 nm, mole fraction (*E*)-**1** (x_E) = [Δ Abs. / Δ Abs. (PSS_{455})] × x_E (PSS_{455}).
b By ^{1}H NMR integration determined as 41:59 (*Z/E*) and thus within ±5% integration error.

^{1}H NMR photoisomerization study of (*Z*)-1

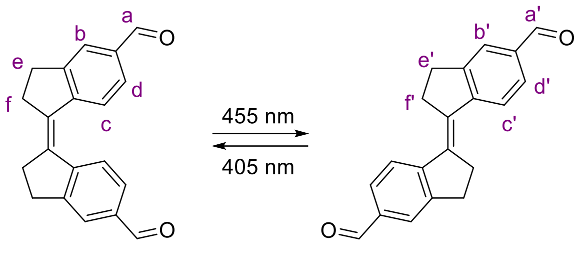

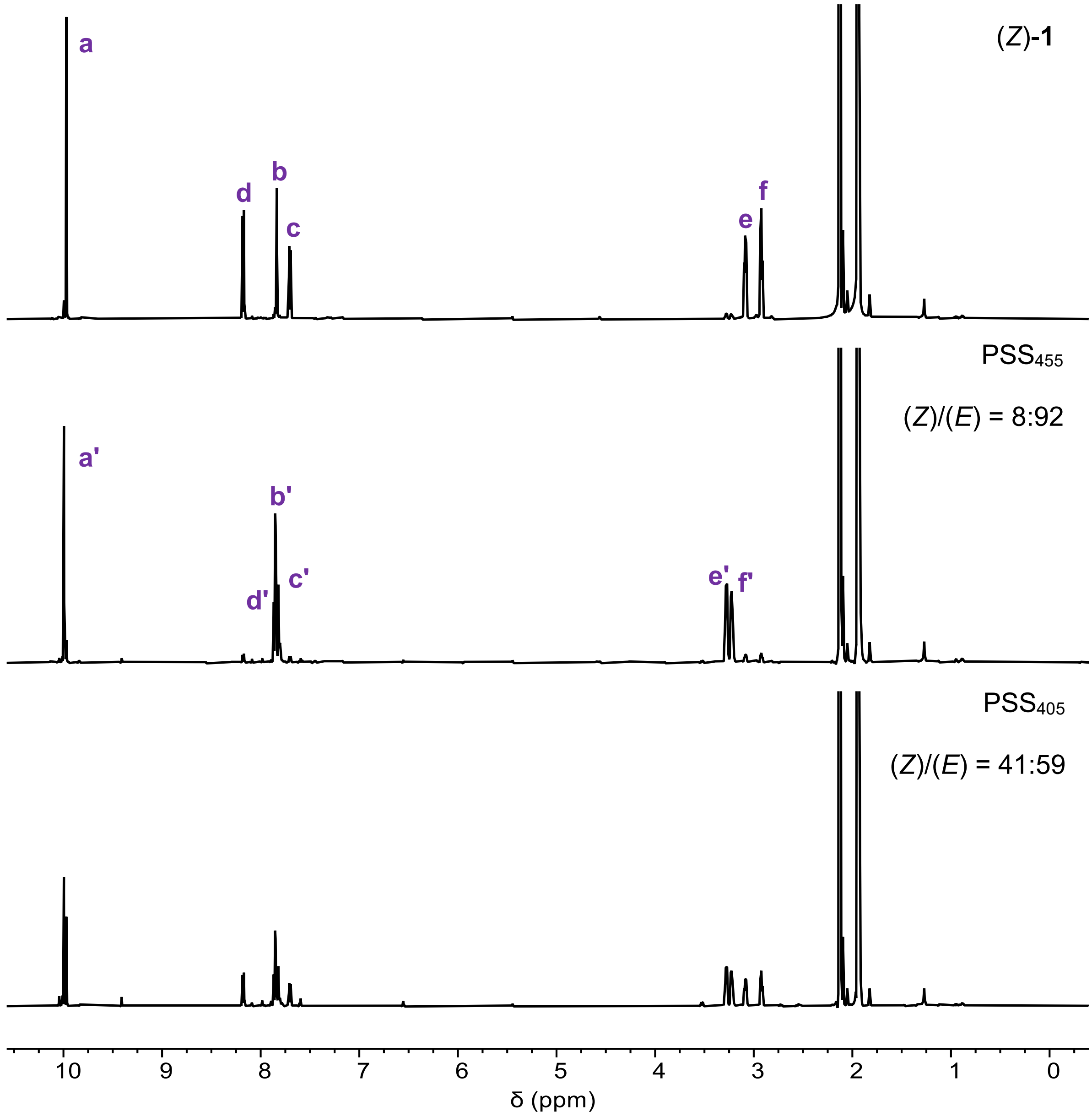

Figure S6. ^{1}H NMR spectrum of (*Z*)-**1** (600 MHz, 0.81 mM in CD_3CN) upon irradiation with 455 nm and 405 nm light. PSS_{455nm} (*Z*/*E*) = 8:92 and PSS_{405nm} (*Z*/*E*) = 41:59 as calculated through integration of H_a, H_e and H_f signals.

^{1}H NMR thermal stability study of 1

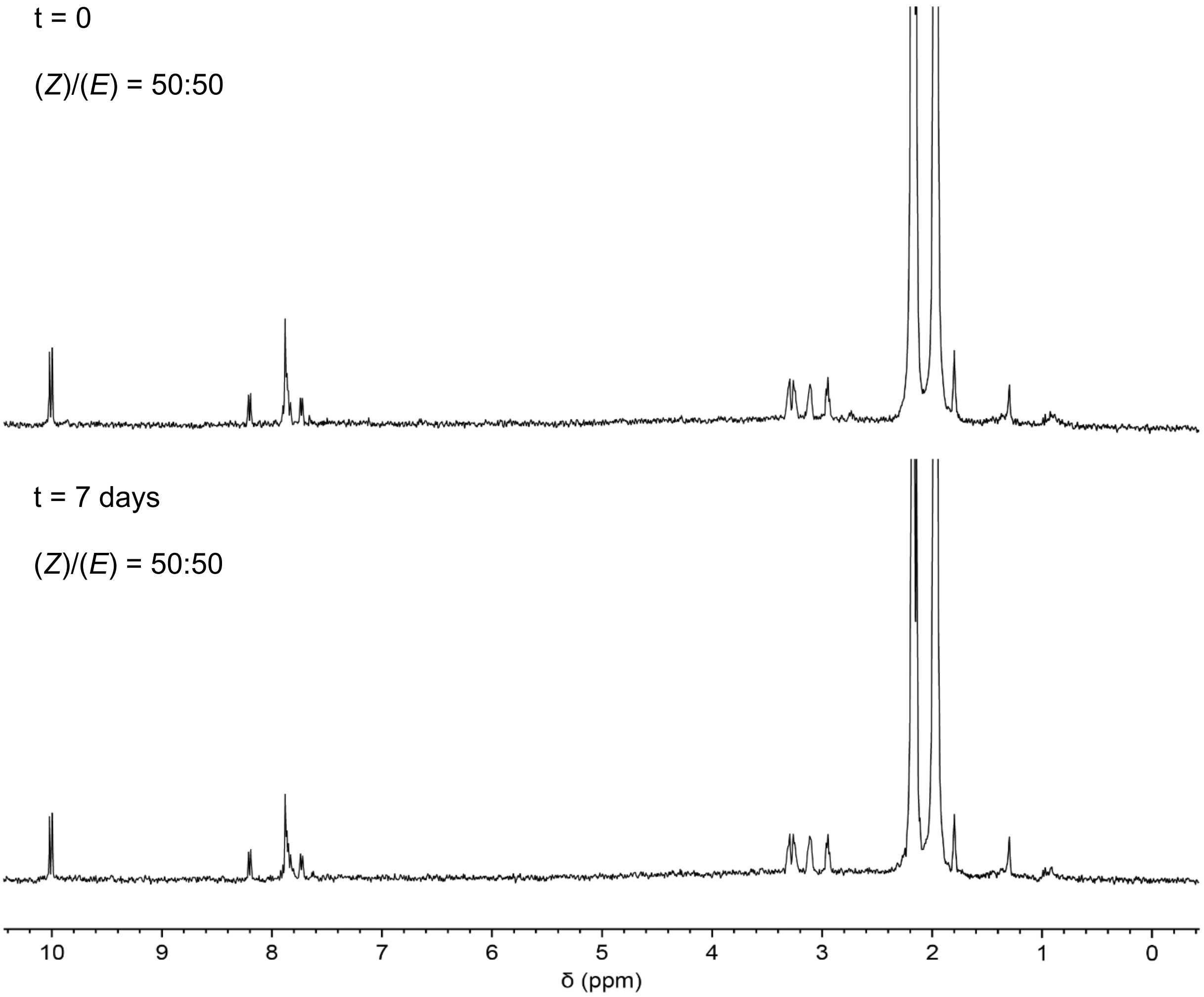

Figure S7. ^{1}H NMR spectrum of a 50:50 (*Z*)-**1**/(*E*)-**1** mixture (400 MHz, 0.80 mM in CD_3CN), obtained by irradiation with 405 nm light (top), and after 7 days at rt in the dark (bottom). The *Z*/*E* ratio was calculated through integration of H_a, H_e and H_f signals. No change in this ratio was observed over time, indicating thermal stability of the photoswitch under these conditions.

^{1}H NMR *in situ* formation study of (*Z*)-2

n-Butylamine (4.2 µL, 42 µmol) was added to (*Z*)-**1** (4.3 mg, 15 µmol) in CD_3OD (1 mL) and the solution was allowed to stand overnight at rt in the dark to give (*Z*)-**2**.

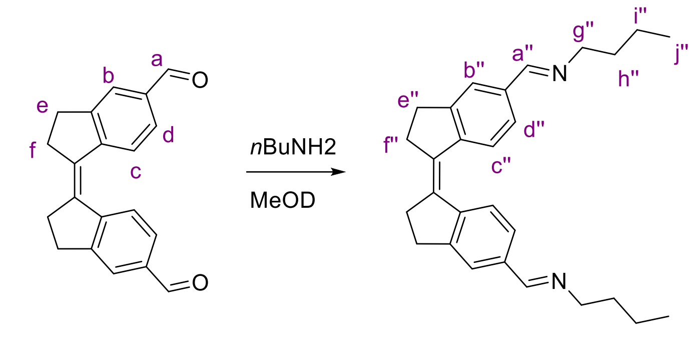

(*Z*)-**1** (*Z*)-**2**

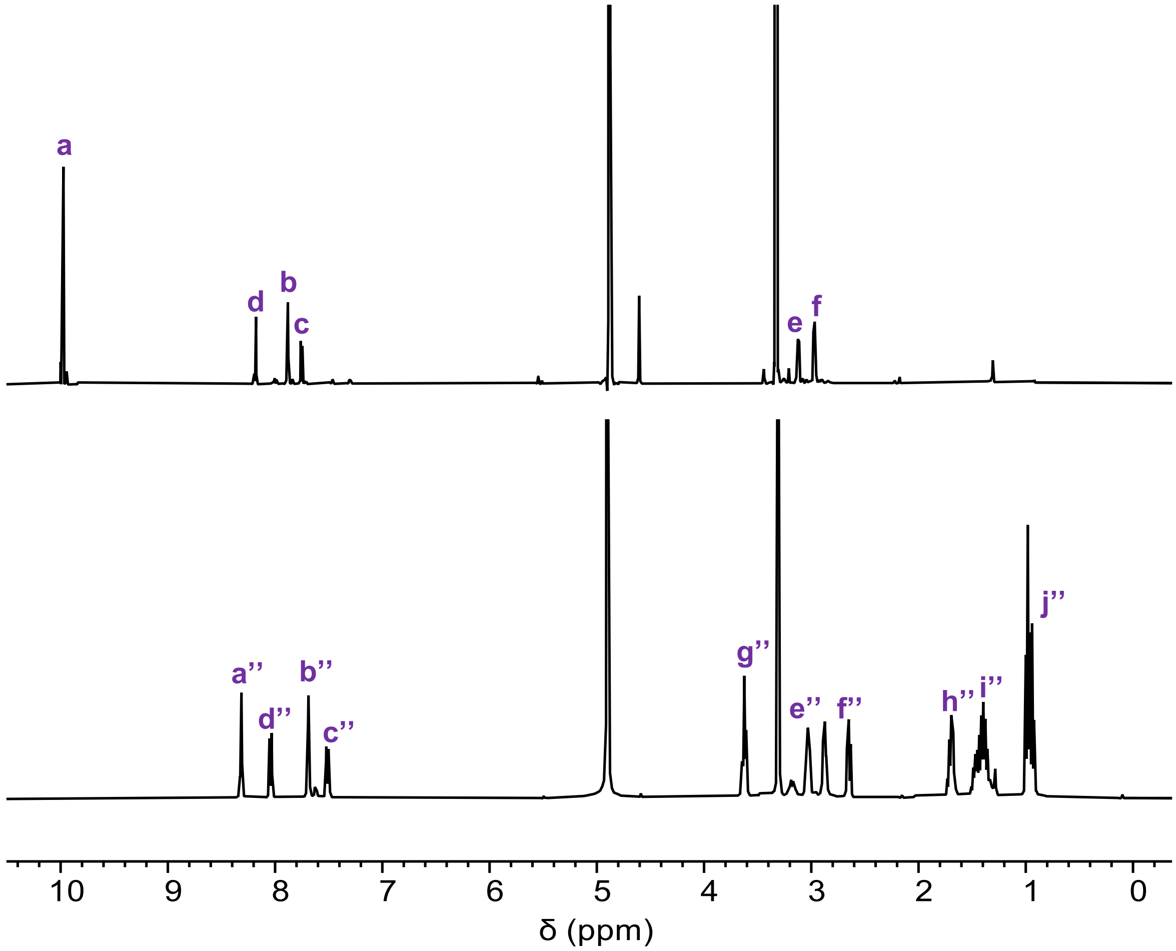

Figure S8. ^{1}H NMR spectrum (400 MHz, 15 mM in CD_3OD) of (*Z*)-**1** without (top) and with (bottom) *n*-butylamine. The spectrum reveals quantitative conversion to (*Z*)-**2**.

UV-vis photoisomerization study of (*Z*)-2

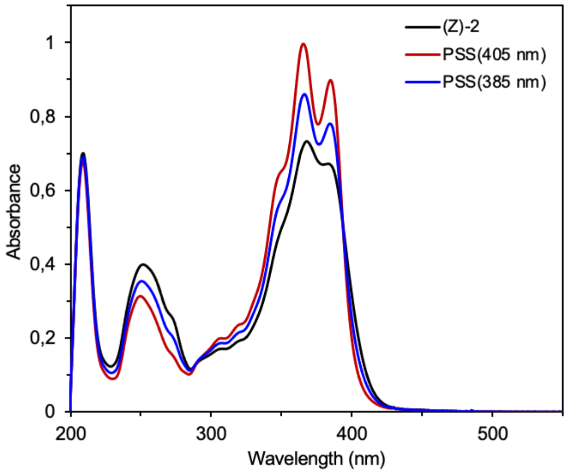

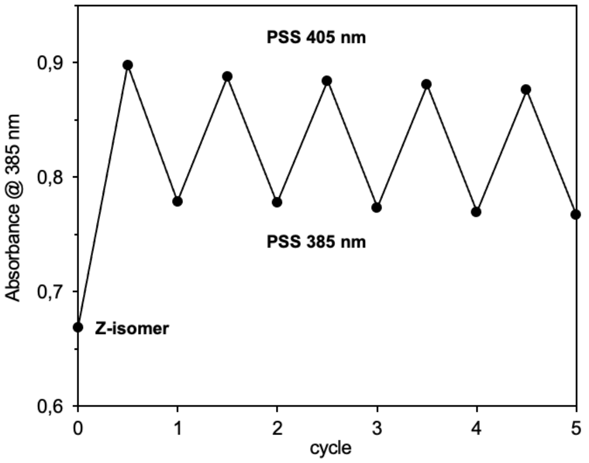

Figure S9. (left) UV-vis absorption spectra of (*Z*)-**2** (2.0 x 10^{-5} M in dry, degassed CH_3CN) upon irradiation with 405 nm and 385 nm and (right) absorbance intensity plot (at $\lambda = 385$ nm) upon sequential irradiation with 405 nm (20 s) and 385 nm (5 s); After five 405/385 nm irradiation cycles, 2% loss in absorbance intensity was observed.

^{1}H NMR study of chemically-controlled photoisomerization

First, a solution of (Z)-**1** (2.0 mM, 1.0 µmol) was prepared in 500 µL CD_3CN/CD_3OD (3:1 v/v) and this solution was irradiated at 455 nm for 22 min until the PSS with a 10:90 *Z*/*E* ratio was reached. Next, the solution was irradiated at 405 nm for 5 min to give a PSS (*Z*/*E*) ratio of 30:70. Then, *n*-butylamine was added (100 µL of 0.1 M in CD_3CN/CD_3OD 3:1 v/v, 10 µmol, 10 equiv.) and the solution stood at rt for 2 days, after which full conversion to the diimine isomers (*Z*)-**2** and (*E*)-**2** was reached, being present in a 30:70 (*Z*/*E*) ratio. Subsequent irradiation at 405 nm for 25 min until PSS was reached gave a change in (*Z*/*E*) ratio to 10:90. Finally, an aqueous acetic acid solution (1M, 50 µL, 50 µmol) was added and the solution was allowed to stand for 3h at rt, after which (*Z*)-**1** and (*E*)-**1** were obtained in a (*Z*/*E*) 10:90 ratio, similar to when the experiment was started. The PSS ratios were determined by relative integration of the aldehyde (for **1**) and imine signals (for **2**).

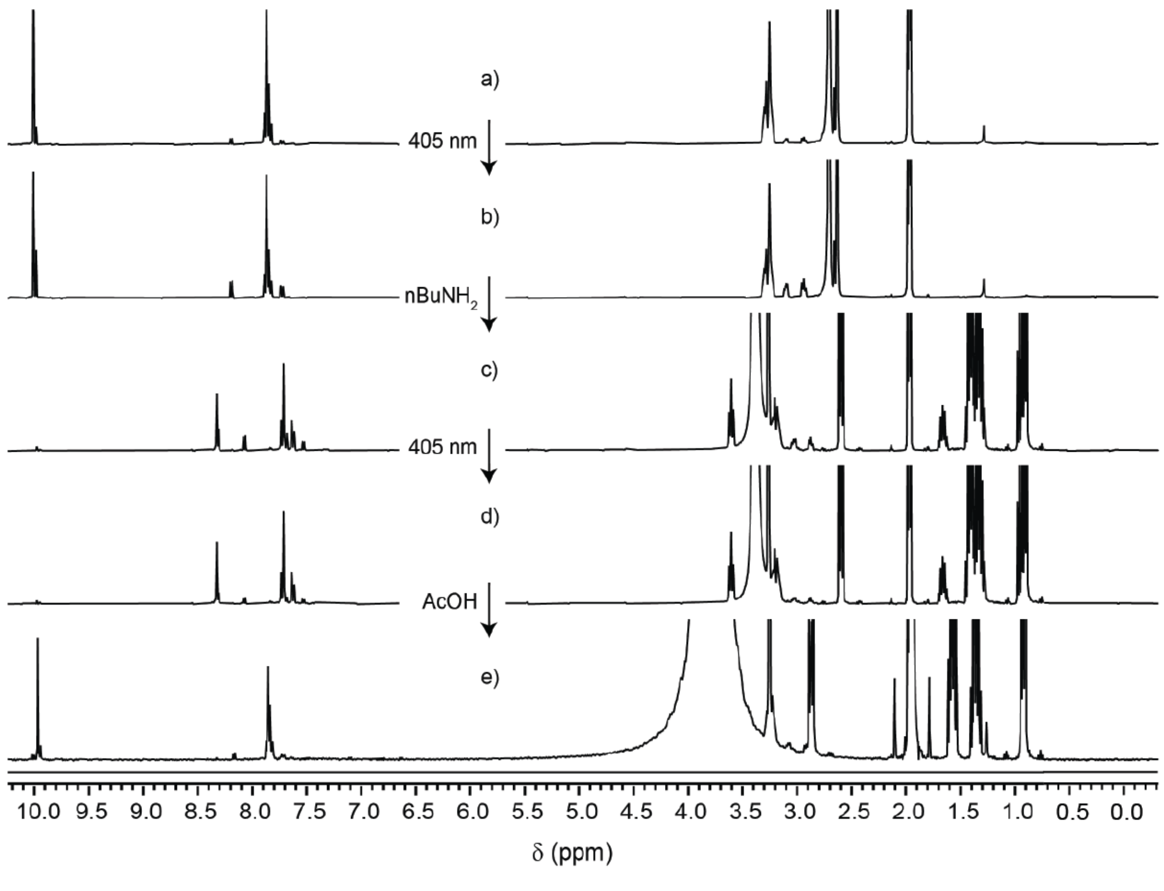

Figure S10. Full ^{1}H NMR spectra (400 MHz, CD_3CN/CD_3OD, 3:1 v/v) of the chemically-controlled photoisomerization cycle using light of one wavelength: a) obtained upon irradiation of (*Z*)-**1** at 455 nm, b) after irradiation at 405 nm, c) after addition of *n*-butylamine, d) after irradiation at 405 nm, and e) after addition of an aqueous acetic acid (AcOH) solution.

Single crystal X-ray crystallography

All reflection intensities were measured at 110(2) K using a SuperNova diffractometer (equipped with Atlas detector) with Mo $K\alpha$ radiation (λ = 0.71073 Å) under the program CrysAlisPro (Version CrysAlisPro 1.171.42.49, Rigaku OD, 2022). The same program was used to refine the cell dimensions and for data reduction. The structure was solved with the program SHELXS-2018/3 (Sheldrick, 2018) and was refined on F^2 with SHELXL-2018/3 (Sheldrick, 2018). Numerical absorption correction based on gaussian integration over a multifaceted crystal model was performed using CrysAlisPro. The temperature of the data collection was controlled using the system Cryojet (manufactured by Oxford Instruments). The H atoms were placed at calculated positions using the instructions AFIX 23 or AFIX 43 with isotropic displacement parameters having values 1.2 U_{eq} of the attached C atoms. The structure is ordered. The molecule is found at one site of inversion symmetry, and thus only one half is found to be crystallographically independent.

Table S2. Crystallographic data of (*E*)-**1**.

	(*E*)-**1**
Crystal data	
Chemical formula	$C_{20}H_{16}O_2$
M_r	288.33
Crystal system, space group	Monoclinic, $P2_1/n$
Temperature (K)	110
a, *b*, *c* (Å)	7.8185 (6), 6.7389 (5), 13.0429 (8)
β (°)	90.468 (6)
V (Å^3)	687.18 (8)
Z	2
Radiation type	Mo *K*α
μ (mm^{-1})	0.09
Crystal size (mm)	0.14 × 0.09 × 0.07
Data collection	
Diffractometer	SuperNova, Dual, Cu at zero, Atlas
Absorption correction	Gaussian<br>*CrysAlis PRO* 1.171.42.49 (Rigaku Oxford Diffraction, 2022) Numerical absorption correction based on gaussian integration over a multifaceted crystal model Empirical absorption correction using spherical harmonics, implemented in SCALE3 ABSPACK scaling algorithm.
T_{min}, T_{max}	0.945, 1.000
No. of measured, independent and observed [$I > 2\sigma(I)$] reflections	7385, 1353, 1032
R_{int}	0.046
$(\sin \theta/\lambda)_{max}$ (Å^{-1})	0.617
Refinement	
$R[F^2 > 2\sigma(F^2)]$, $wR(F^2)$, S	0.042, 0.107, 1.03
No. of reflections	1353
No. of parameters	100
H-atom treatment	H-atom parameters constrained
$\Delta\rho_{max}$, $\Delta\rho_{min}$ (e Å^{-3})	0.19, -0.21

Computer programs: *CrysAlis PRO* 1.171.42.49 (Rigaku OD, 2022), *SHELXS2018*/3 (Sheldrick, 2018), *SHELXL2018*/3 (Sheldrick, 2018), *SHELXTL* v6.10 (Sheldrick, 2008).[7]

Time-dependent DFT calculations

Calculations were performed using Gaussian 09.[8] Geometry optimizations were performed at the B3LYP/6-311++G(d,p) level of theory using and IEFPCM CH_3CN solvation model. All computed stationary points were subjected to frequency analysis and confirmed as local minima. Using the DFT geometry-optimized structures, TD-DFT calculations (solving for 30 singlet excited states) were performed using the same level of theory and solvation model. To reduce computational costs, the butyl-chain for the diimine derivatives was shortened to ethyl.

Table S3. Cartesian coordinates of (*Z*)-**1**.

Atom	X	Y	Z
C	-0.49438	2.90164	2.44278
H	-1.58102	3.00153	2.54379
H	-0.04091	3.70579	3.02533
C	-0.03656	1.49322	2.89059
H	0.95750	1.55384	3.35078
H	-0.70654	1.04871	3.62748
C	0.03656	0.67734	1.60228
C	0.18933	1.64933	0.50906
C	-0.09781	2.94499	0.98805
C	0.66819	1.48751	-0.80388
H	0.96588	0.51485	-1.17019
C	0.79900	2.59384	-1.62741
H	1.17434	2.48342	-2.63781
C	0.46396	3.87841	-1.16284
C	0.02345	4.04941	0.15871
H	-0.20573	5.04544	0.52555
C	0.59350	5.05938	-2.02874
H	0.32115	6.01777	-1.54597
O	0.96465	5.04498	-3.19131
C	0.49438	-2.90164	2.44278
H	0.04091	-3.70579	3.02533
H	1.58102	-3.00153	2.54379
C	0.03656	-1.49322	2.89059
H	0.70654	-1.04871	3.62748
H	-0.95750	-1.55384	3.35078
C	-0.03656	-0.67734	1.60228
C	-0.18933	-1.64933	0.50906
C	0.09781	-2.94499	0.98805
C	-0.66819	-1.48751	-0.80388
H	-0.96588	-0.51485	-1.17019
C	-0.79900	-2.59384	-1.62741
H	-1.17434	-2.48342	-2.63781
C	-0.46396	-3.87841	-1.16284
C	-0.02345	-4.04941	0.15871
H	0.20573	-5.04544	0.52555
C	-0.59350	-5.05938	-2.02874
H	-0.32115	-6.01777	-1.54597
O	-0.96465	-5.04498	-3.19131

Table S4. Cartesian coordinates of (*E*)-**1**.

Atom	X	Y	Z
C	-1.81006	2.45327	0.47247
H	-1.81403	2.70659	1.53820
H	-2.13071	3.34612	-0.06834
C	-0.41288	1.95657	0.03111
H	-0.16688	2.36627	-0.95505
H	0.36972	2.27955	0.71858
C	-0.52089	0.43638	-0.04473
C	-1.95404	0.11247	-0.06090
C	-2.70953	1.27171	0.21632
C	-2.62161	-1.09629	-0.34198
H	-2.07576	-1.99236	-0.59732
C	-4.00711	-1.13513	-0.32232
H	-4.53120	-2.05717	-0.54411
C	-4.75459	0.01790	-0.02703
C	-4.09440	1.22765	0.23989
H	-4.67324	2.12017	0.45809
C	-6.22436	-0.01123	-0.00393
H	-6.70140	0.96005	0.23008
O	-6.91444	-0.99503	-0.21668
C	1.81000	-2.45322	0.47247
H	2.13064	-3.34608	-0.06833
H	1.81395	-2.70654	1.53820
C	0.41284	-1.95650	0.03109
H	-0.36978	-2.27947	0.71854
H	0.16685	-2.36619	-0.95508
C	0.52089	-0.43631	-0.04473
C	1.95404	-0.11243	-0.06091
C	2.70951	-1.27169	0.21633
C	2.62166	1.09630	-0.34201
H	2.07583	1.99239	-0.59737
C	4.00716	1.13510	-0.32234
H	4.53127	2.05713	-0.54415
C	4.75460	-0.01794	-0.02703
C	4.09437	-1.22766	0.23991
H	4.67319	-2.12020	0.45812
C	6.22438	0.01115	-0.00392
H	6.70138	-0.96014	0.23013
O	6.91448	0.99493	-0.21666

Table S5. Cartesian coordinates of (*Z*)-**2**.

Atom	X	Y	Z
C	-2.85008	-3.27254	-0.73372
H	-2.85636	-3.37923	-1.82474
H	-3.68957	-3.85535	-0.34889
C	-1.48574	-3.71692	-0.15487
H	-1.63310	-4.17161	0.83293
H	-0.98709	-4.45906	-0.77976
C	-0.67759	-2.42715	-0.01980
C	-1.66312	-1.33470	0.04267
C	-2.92841	-1.81548	-0.34843
C	-1.55439	-0.01687	0.51951
H	-0.61300	0.36121	0.89419
C	-2.67307	0.80308	0.54946
H	-2.59386	1.81648	0.92356
C	-3.92706	0.33762	0.11358
C	-4.04409	-0.98977	-0.32655
H	-5.01333	-1.36937	-0.63633
C	-5.12654	1.18985	0.12801
H	-6.05278	0.69498	-0.20046
N	-5.12784	2.41162	0.48644
C	2.85008	-3.27254	0.73370
H	3.68957	-3.85535	0.34886
H	2.85636	-3.37924	1.82472
C	1.48574	-3.71692	0.15485
H	0.98709	-4.45907	0.77973
H	1.63310	-4.17160	-0.83296
C	0.67759	-2.42715	0.01978
C	1.66312	-1.33470	-0.04267
C	2.92841	-1.81548	0.34842
C	1.55438	-0.01687	-0.51951
H	0.61300	0.36122	-0.89419
C	2.67307	0.80308	-0.54945
H	2.59386	1.81648	-0.92355
C	3.92705	0.33762	-0.11358
C	4.04408	-0.98977	0.32655
H	5.01333	-1.36938	0.63632
C	5.12654	1.18985	-0.12800
H	6.05278	0.69498	0.20046
N	5.12784	2.41162	-0.48643
C	-6.39295	3.13474	0.44686
C	-6.29918	4.36125	-0.46331
H	-6.61568	3.46541	1.46792
H	-7.22025	2.48509	0.12313
H	-5.49414	5.02553	-0.13899
H	-7.23769	4.92148	-0.44093
H	-6.10236	4.06433	-1.49705
C	6.39295	3.13475	-0.44685
C	6.29919	4.36125	0.46334
H	6.61568	3.46541	-1.46790
H	7.22025	2.48509	-0.12311
H	5.49414	5.02553	0.13902
H	7.23770	4.92148	0.44095
H	6.10236	4.06432	1.49707

Table S6. Cartesian coordinates of (*E*)-**2**.

Atom	X	Y	Z
C	1.64206	2.56846	-0.62806
H	1.62615	2.82197	-1.69402
H	1.90220	3.48203	-0.08860
C	0.28210	1.97854	-0.18379
H	0.01218	2.36881	0.80429
H	-0.52366	2.25108	-0.86685
C	0.49151	0.46828	-0.11286
C	1.94486	0.24405	-0.09743
C	2.62033	1.44957	-0.37336
C	2.70136	-0.91085	0.17915
H	2.22337	-1.84610	0.43274
C	4.08791	-0.85101	0.15767
H	4.67221	-1.73616	0.37750
C	4.76003	0.34833	-0.13602
C	4.00650	1.50312	-0.39964
H	4.51502	2.43776	-0.61771
C	6.22872	0.43551	-0.16413
H	6.63172	1.42961	-0.40882
N	7.00458	-0.54633	0.07074
C	-1.64206	-2.56846	-0.62807
H	-1.90220	-3.48203	-0.08861
H	-1.62615	-2.82196	-1.69402
C	-0.28210	-1.97855	-0.18379
H	0.52366	-2.25108	-0.86685
H	-0.01218	-2.36882	0.80429
C	-0.49151	-0.46828	-0.11286
C	-1.94486	-0.24405	-0.09743
C	-2.62033	-1.44957	-0.37337
C	-2.70136	0.91085	0.17916
H	-2.22336	1.84610	0.43274
C	-4.08791	0.85101	0.15767
H	-4.67221	1.73616	0.37751
C	-4.76003	-0.34833	-0.13602
C	-4.00650	-1.50312	-0.39965
H	-4.51502	-2.43776	-0.61772
C	-6.22872	-0.43550	-0.16413
H	-6.63172	-1.42961	-0.40882
N	-7.00458	0.54634	0.07074
C	-8.44210	0.31157	0.01069
C	-9.11271	0.65098	1.34366
H	-8.67058	-0.72643	-0.27494
H	-8.85049	0.96430	-0.76944
H	-10.19565	0.52328	1.26545
H	-8.90819	1.68588	1.62961
H	-8.74770	-0.00272	2.14048
C	8.44210	-0.31156	0.01069
C	9.11271	-0.65098	1.34366
H	8.85049	-0.96430	-0.76945
H	8.67058	0.72644	-0.27494
H	10.19565	-0.52328	1.26545
H	8.90819	-1.68588	1.62961
H	8.74770	0.00272	2.14048

	Symmetry	eV	nm	Oscillator strength	<S^2>	coefficients
(*E*)-**1**	A	3.01	411.63	1.1713	0.000	0.70694
(*Z*)-**1**	B	2.96	418.36	0.8142	0.000	0.70695

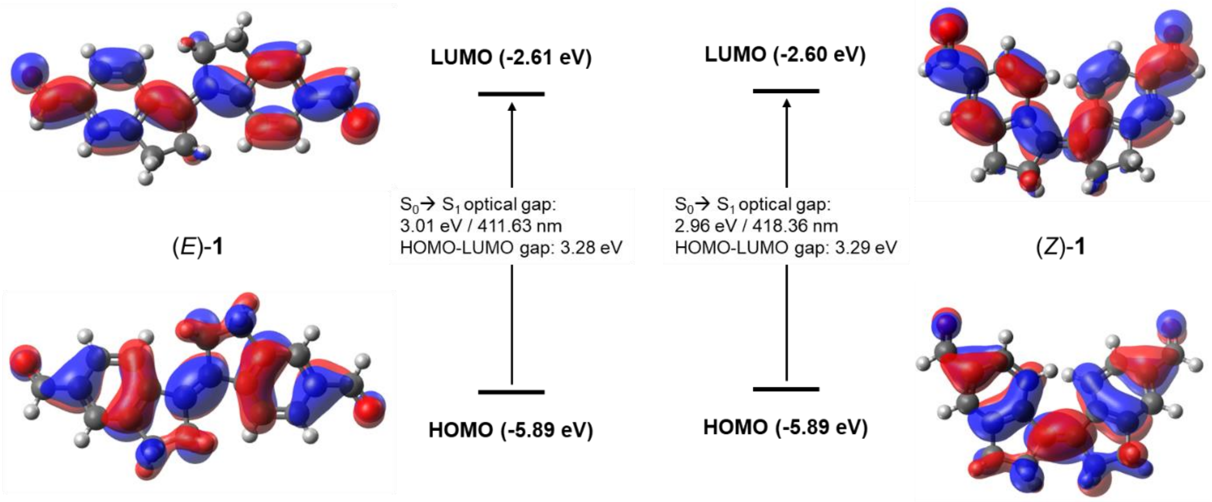

Figure S11. Description and visualization of first excited state of (*E*)-**1** and (*Z*)-**1** described by an excitation from HOMO (molecular orbital 76) to LUMO (molecular orbital 77).

	Symmetry	eV	nm	Oscillator strength	<S^2>	coefficients
(*E*)-**2**	A	3.11	398.30	1.5587	0.000	0.70613
(*Z*)-**2**	A	3.06	404.58	0.9831	0.000	0.70612

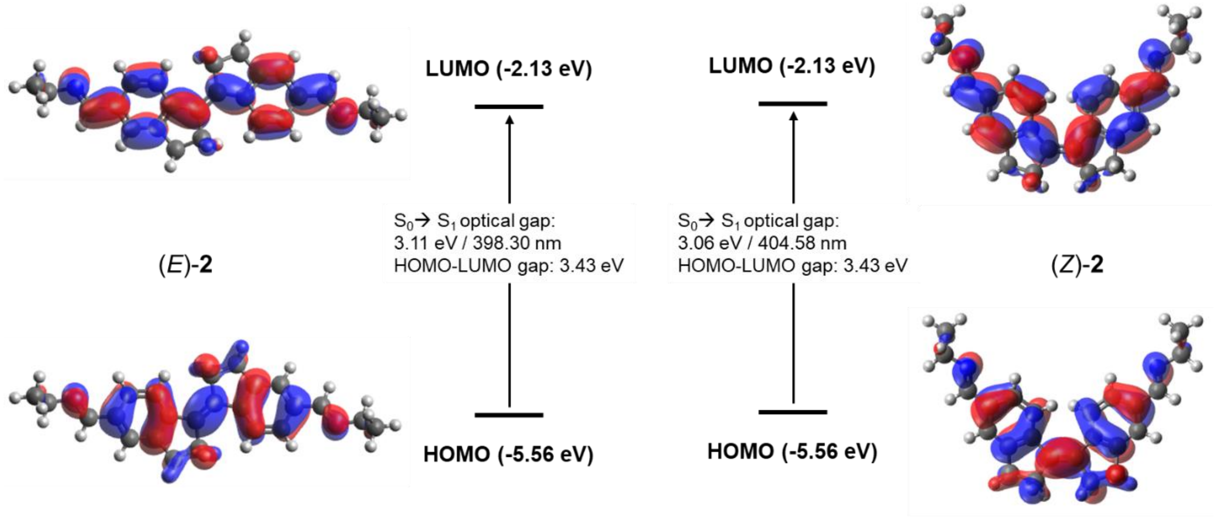

Figure S12. Description and visualization of first excited state of (*E*)-**2** and (*Z*)-**2** described by an excitation from HOMO (molecular orbital 92) to LUMO (molecular orbital 93).

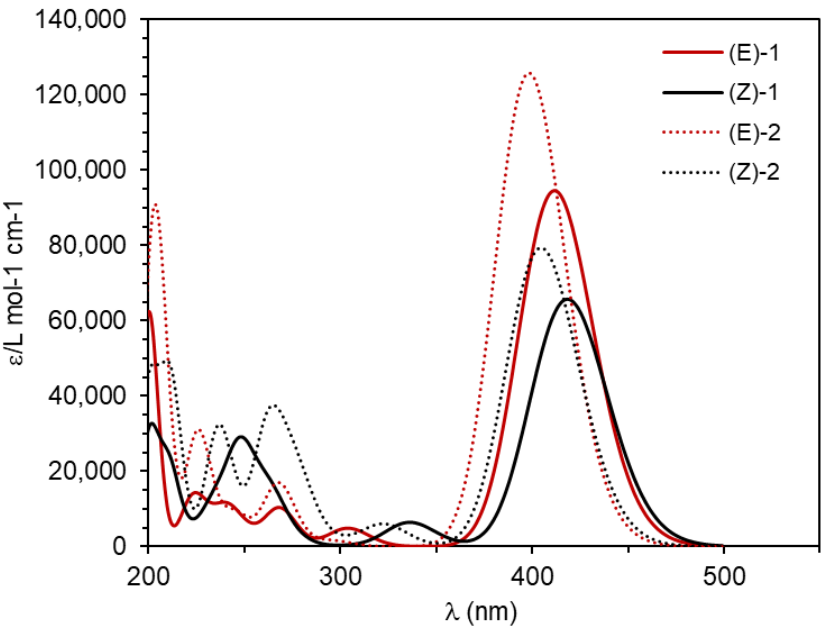

Figure S13. Computed UV-vis absorption spectra of (*E*)-**1**, (*Z*)-**1**, (*E*)-**2** and (*Z*)-**2**, plotted with peak half-width at half height set to 0.167 eV.

References

(1) Villarón, D.; Duindam, N.; Wezenberg, S. J. Push-Pull Stiff-Stilbene: Proton-Gated Visible-Light Photoswitching and Acid-Catalyzed Isomerization. *Chem. Eur. J.* **2021**, *27*, 17346–17350.

(2) Liang, A.; Huang, G.; Wang, Z.; Wu, W.; Zhong, Y.; Zhao, S.; Cao, R.; Chen, S.; Hou, H. Supramolecular Green Phosphorescent Polymer Iridium Complexes for Solution-Processed Nondoped Organic Light-Emitting Diodes. *J. Organomet. Chem.* **2016**, *804*, 1–5.

(3) Egert, M.; Walther, S.; Plenio, H. Synthesis of Substituted Imidazolidines: Base-Stable Precursors of 4,5-Dihydro-1 H -imidazol-3-ium Salts and N -Heterocyclic Carbenes. *Eur. J. Org. Chem.* **2014**, *2014*, 4362–4369.

(4) Murai, M.; Enoki, T.; Yamaguchi, S. Dithienoazepine-Based Near-Infrared Dyes: Janus-Faced Effects of a Thiophene-Fused Structure on Antiaromatic Azepines. *Angew. Chem. Int. Ed.* **2023**, *62*, e202311445.

(5) Li, T.-Y.; Lin, Y.-C.; Song, Y.-H.; Lu, H.-F.; Chao, I.; Lin, C.-H. Synthesis and Physical Study of Perylene and Anthracene Polynitrile as Electron Acceptors. *Org. Lett.* **2019**, *21*, 5397–5401.

(6) Jin, Y.; Ju, J.; Kim, J.; Lee, S.; Kim, J. Y.; Park, S. H.; Son, S.-M.; Jin, S.-H.; Lee, K.; Suh, H. Design, Synthesis, and Electroluminescent Property of CN−Poly(Dihexylfluorenevinylene) for LEDs. *Macromolecules* **2003**, *36*, 6970–6975.

(7) Sheldrick, G. M. Crystal Structure Refinement with SHELXL. *Acta Crystallogr. Sect. C Struct. Chem.* **2015**, *71*, 3–8.

(8) Frisch, M. J.; Trucks, G. W.; Schlegel, H. B.; Scuseria, G. E.; Robb, M. A.; Cheeseman, J. R.; Scalmani, G.; Barone, V.; Petersson, G. A.; Nakatsuji, H.; Li, X.; Caricato, M.; Marenich, A.; Bloino, J.; Janesko, B.; Gomperts, G.; Mennucci, R. B.; Hratchian, H. P.; Ort, J. V.; Ochterski, J. W.; Martin, R. L.; Morokuma, K.; Farkas, O.; Foresman, J. B.; Fox, D. J. Gaussian 09, Revision A.02. Gaussian, Inc.: Wallingford CT 2016.
